# Tillage System and Crop Sequence Affect Soil Disease Suppressiveness and Carbon Status in Boreal Climate

**DOI:** 10.3389/fmicb.2020.534786

**Published:** 2020-10-23

**Authors:** Ansa Palojärvi, Miriam Kellock, Päivi Parikka, Lauri Jauhiainen, Laura Alakukku

**Affiliations:** ^1^Natural Resources Institute Finland (Luke), Turku, Finland; ^2^Natural Resources Institute Finland (Luke), Jokioinen, Finland; ^3^Department of Agricultural Sciences, University of Helsinki, Helsinki, Finland

**Keywords:** fungistasis, no-till, non-inversion, *Fusarium* spp., microbial biomass, general disease suppression, crop rotation, labile carbon

## Abstract

The soil-borne plant pathogens cause serious yield losses and are difficult to control. In suppressive soils, disease incidence remains low regardless of the presence of the pathogen, the host plant, and favorable environmental conditions. The potential to improve natural soil disease suppressiveness through agricultural management practices would enable sustainable and resilient crop production systems. Our aim was to study the impact of autumn tillage methods and crop sequence on the soil carbon status, fungistasis and yield in boreal climate. The disease suppression was improved by the long-term reduced and no tillage management practices with and without crop rotation. Compared to the conventional plowing, the non-inversion tillage systems were shown to change the vertical distribution of soil carbon fractions and the amount of microbial biomass by concentrating them on the soil surface. Crop sequence and the choice of tillage method had a combined effect on soil organic carbon (SOC) sequestration. The improved general disease suppression had a positive correlation with the labile carbon status and microbial biomass. From the most common *Fusarium* species, the predominantly saprophytic *F. avenaceum* was more abundant under non-inversion practice, whereas the opposite was true for the pathogenic ones. Our findings furthermore demonstrated the correlation of the soil fungistasis laboratory assay results and the prevalence of the pathogenic test fungus *Fusarium culmorum* on the crop cereals in the field. Our results indicate that optimized management strategies have potential to improve microbial related soil fungistasis in boreal climate.

## Introduction

Diseases caused by soil-borne pathogens are among the most important limiting factors for plant growth and productivity (Oerke, 2006). Due to the pathogens abilities to survive in the soil for long periods of time, even without the plant host, they cause problems worldwide (Wang and Li, 2019). Some of the soil-borne fungal pathogens produce mycotoxins, such as deoxynivalenol (DON) by *Fusarium* spp. (Parikka et al., 2012; Hietaniemi et al., 2016; Hofgaard et al., 2016). In modern farming, simple crop sequence, conventional tillage, and cultivation of only a limited number of crop varieties favor the increased incidence and severity of diseases caused by necrotrophic soil-borne pathogens (Cha et al., 2016).

The overall aim of sustainable farming is to cut down the use of pesticides (European Commission, 2017). The greater reliance on the beneficial functions and ecosystem services provided by the soil microbiome is a promising approach forward (Sipilä et al., 2012; Constanzo and Barberi, 2014; de Boer et al., 2019). Disease suppressive soils, through the competitive activity of the non-pathogenic residents of the total soil microbiota (general suppression) or the antagonistic capabilities of specific groups of microorganisms (specific suppression), are able to reduce the occurrence or severity of diseases caused by soil-borne phytopathogens (Weller et al., 2002; Dignam et al., 2018). For most soil pathogens, however, the microorganisms responsible for suppression and the suppression mechanisms are not fully known, but it is likely that the soil suppressiveness is a mixture of both types of suppressiveness (Postma et al., 2008). The complex interplay of soil suppressiveness cannot simply relate to a single microbial taxon or group (de Boer et al., 2007; Legrand et al., 2019). The majority of rhizobacterial taxa indicative of the suppressiveness status of the soil may differ when comparing different types of suppressive soils or even different soils suppressive to a same phytopathogen (de Boer et al., 2019; Wang and Li, 2019).

Fungistasis is one form of soil suppressiveness, defined as the ability of the soil to restrict the germination and growth of fungi (Lockwood, 1977; Garbeva et al., 2011). The key mechanism that explains soil fungistasis is intensive competition for nutrients within the soil microbial community. Along with this, the production of antifungal compounds in different forms, including volatile organic compounds (VOC), may play a major role (Garbeva et al., 2011; van Agtmaal et al., 2018). Most likely suppressive soils are governed by microbial consortia where saprotrophic fungi have an important role (Mendes et al., 2011; Sipilä et al., 2012; Penton et al., 2014; van Agtmaal et al., 2017).

On global scale, conservation agriculture with either reduced or no tillage management, crop rotation and crop cover has increased rapidly during the last decades and was evaluated to be ca. 180 million ha in 2016 (Prestele et al., 2018; Kassam et al., 2019). Crop residues left untouched, as happens in non-inversion management, accumulates organic matter on the surface layer of soil (Muukkonen et al., 2007; Singh et al., 2015; Laine et al., 2018; Ogle et al., 2019). The carbon allocation, mixing intensity, and soil moisture and temperature conditions, affect the distribution and living conditions of microbial communities in soil. Soil and crop residue-borne plant pathogens have been reported to benefit from crop residues on the soil surface (Hofgaard et al., 2016). On the other hand, reduced tillage practices, crop species selection, diverse crop rotation and practices to increase organic matter in soils are all shown to increase the amount of microbial biomass in soil, and also tend to improve disease suppressive activity of soil (Janvier et al., 2007; Sipilä et al., 2012). However, the underlying mechanisms and the relationship between disease suppression and agrotechnological practices are still not fully understood.

In their cross-site study of soil microbial communities and *Fusarium* sp. fungistasis on long-term no-till and moldboard plowed treatments, Sipilä et al. (2012) ended up to a general model of interlink between low and high amount of organic matter resources for microbial metabolism together with microbial biomass and interactions. Labile carbon has been shown to be a sensitive soil quality indicator for impacts of tillage and organic matter inputs on microbial pools and activity (Bongiorno et al., 2019a,b). More studies comparing the different carbon pools [Soil Organic Carbon (SOC), labile C, microbial biomass C] of soil and their contribution to the general disease suppressiveness are needed. Also, the prevalence of the actual model disease for suppression on the field needs to be studied. The emergence of soil-borne plant diseases is a result of the interactions between micro-organisms, pathogens and plants in the complex physical environment of soil. Farming practices that affect these soil qualities have potential to influence the general suppressiveness of soil against soil and residue-borne plant pathogens, and further, the crop yield. More insight is needed on the impact of reduced tillage practice and use of crop sequence.

The objectives of this study were to examine the impact of autumn tillage methods (no-till, stubble cultivation, and plowing) and crop sequence on the soil carbon status and the development of the general plant pathogen suppressiveness (test species pathogenic *Fusarium culmorum* fungus). The occurrence of soil and plant residue transmitted plant diseases (*Fusarium* spp.) were studied in a long term experimental field with two different crop sequences [spring barley (*Hordeum vulgare* L.) monoculture, 4-year crop rotation] in boreal climate.

## Materials and Methods

### Field Site and Sampling

A long-term experimental field, located in Jokioinen in southwest Finland (coordinates 60°49′N, 23°28′E), was used in the study [Regina and Alakukku, 2010 (site 2); Sipilä et al., 2012 (site 2)]. The field consists of clay soil, 0-20 cm layer with a mean clay content of 62% and 20-40 cm layer with a mean clay content of 81%, and is classified as a Vertic Endostagtic Cambisol (IUSS Working Group WRB, 2006). The field experiment was established in year 2000 to compare different primary tillage treatments: (i) autumn plowing (mouldboard plowed about 20-23 cm depth), (ii) reduced tillage (autumn stubble cultivation 10-12 cm), and (iii) no-till (direct drilling in spring). Since 2011, two crop sequences were established as sub-plots to the main plots: (i) Spring barley (*Hordeum vulgare*) monoculture was continued, and (ii) a 4 year crop rotation system was started: spring barley (2011), faba bean (*Vicia faba*) (2012), spring oats (*Avena sativa*) (2013), spring turnip rape (*Brassica rapa subsp. oleifera*; 2014). The split-plot experimental design contained two factors: tillage (main-plot factor) and crop sequence (split-plot factor). Four replicates were divided into three main plots each containing two sub-plots (split plots). The three levels of the tillage factor (plowing, reduced, and no-tillage) were randomized to the main plots. The two levels of crop sequence factor (monoculture, crop rotation) were randomized to sub-plots within each three main plot. The randomizations were repeated at the four replications separately. Each sub-plot (split plot) was 9 m wide and 40 m long, a total 360 m^2^.

In spring, autumn tilled treatments were sown by combined rotary harrow and drill (one-pass method, combined drill). The seedbed was prepared to 5 cm depth. No-tilled treatment was directly sown to 3–5 cm depth with combined drill having double disk coulters. Mineral fertilizers were used for barley 90 N, 3.3 K kg/ha, for faba bean 30 N, 1.1 K kg/ha, and for oats 60 N, 2.2 K kg/ha (reduced amount due to presiding N-fixing faba bean). Weather parameters in study year 2013 (mean of years 2003-2012 in parenthesis) were: annual precipitation 562 mm (656 mm), annual mean air temperature 5.6°C (5.1°C), number of temperature days where ground temperature minimum >0.0°C 195 (184).

Composite soil samples (minimum 20 subsamples with the augers of diameter of 2 cm) from each treatment plot were randomly collected in October 2013 before tillage. The soils were sampled at depths of 0–5, 5-10, and 10–20 cm, and manually homogenized on site. Samples were divided for air drying, cold room storage (+4°C in dark) and freezer (−18°C).

### Physical and Chemical Analyses of Soil

The physical and chemical properties of the soil samples were analyzed as described in Regina and Alakukku (2010) and Singh et al. (2015). Soil pH and electrical conductivity (EC) were measured in water suspensions (1:2.5 v/v). Inorganic nitrogen (NH_4_-N + NO_3_-N; N_*min*_) in soil was extracted with 1 M KCl (v/v 1:2.5) and analyzed by Skalar autoanalyzer (SKALAR SA 40 5101). Soluble reactive phosphorus (P_*Acetate*_) [extraction with 0.5 M acid ammonium acetate, pH 4.65, Vuorinen and Mäkitie (1955)] was analyzed by spectrophotometry by molybdenum blue method.

### Soil Carbon Fractions

The soil organic carbon (SOC) and total nitrogen (N_*tot*_) content was determined from air dried, ground samples sieved through a 2 mm sieve and analyzed using the Leco CN-2000 analyzer (LECO, St. Joseph, MI, United States). Particulate Organic Matter Carbon (POM-C), which is the labile soil carbon fraction most available for microbes, was analyzed based on wet sieving (Cambardella and Elliott, 1992). The dry bulk density of soil was determined using the volume accurate Kopec corer with a diameter of 5 cm for sampling (three subsamples per plot).

The soil microbial biomass carbon (C_*mic*_) was measured by chloroform fumigation extraction method, for which the total soluble organic carbon was determined from the 0.5 M K_2_SO_4_ extracts using a Shimadzu TOC-V CSH Total organic carbon analyzer. Results are given as soil oven-dry basis (105°C, 48 h).

The total amount of SOC and C_*mic*_ in the top soil layer was calculated two ways: by using the content of SOC and C_*mic*_ on top soil layers (0-20 cm) per m^2^ and by using the equivalent mineral soil mass method (200 kg DM soil; soil layer corresponding approx. 0-15 cm) to compare the total soil C stock between the treatments (Ellert and Bettany, 1995; Wendt and Hauser, 2013; Singh et al., 2015).

### Fungistasis Surface Bioassay

Fungistasis bioassay was performed using the surface method described by de Boer et al. (1998) and Sipilä et al. (2012), with slight modifications. Three replicates were measured. *Fusarium culmorum* was used as inoculum in fungistasis experiment because of its relevance as a common pathogen of barley and oats with soil and plant residue related dispersion (Knudsen et al., 1999). Fungistasis plates were prepared by diluting the test soil from the experimental field (soil depth 0–5 cm) with a sterile clay-sand mixture. Sterile kaolin clay (Quality China Clay, Imerys) and sand (0.2–1 mm) (50/50%) was used as diluent and in the control petri dishes included in each assay. The soil 10/90% dilution level was selected on basis of preliminary experiments. The water content of each plate was adjusted to 75% of water holding capacity of the soil mixture (fresh weight 50 g). Freshly grown *F. culmorum* [from the growth margin on potato dextrose agar (PDA) medium] was used in the bioassay as 1.5 cm diameter circle and inoculated on sterile cover glass (diam. 1.8 cm), placed on the test soil (50 g) in a petri dish (9 cm). The petri dishes were sealed with double wrapping of parafilm and incubated 7 days at 20°C. The area (cm^2^) of fungal growth was measured using microscopic photography. The average extension in sterile kaolin clay and sand mixture (control) was 10.8 cm^2^.

### *Fusarium* spp. Observations From the Crops in the Field

*Fusarium* spp. contamination of developing grain was investigated three times during the growth period. Samples from the stem base were taken 2 weeks after the heading phase. To investigate *Fusarium* species on stem bases, whole plants were sampled (50 stems per sample) and 1 cm pieces of stem bases were incubated on PCNB medium (Pentachloronitrobenzene; Nash and Snyder medium; Nelson et al., 1983) at 22°C. At the same way, *Fusarium* species on stubble were investigated after harvest. *Fusarium* contamination of harvested, dried grain was determined of 100 grains/sample.

The resulting colonies were inoculated for identification on PDA medium and cultured in the dark. *Fusarium* species were determined from cultures using the microscope, and contamination% values for each species were counted of the identified colonies. The most common species were the toxins forming pathogens *F. culmorum* (FC) and *F. graminearum*, (FG) and predominantly saprophytic species *F. avenaceum* (FA).

### Statistical Analysis

Statistical analysis based on a split-plot experimental design (Box et al., 2005) where main plots included three different tillage treatments, while two different crop sequence systems (monoculture, and the 4 year crop rotation-system) were randomly assigned to sub-plots. The experiment included four replications (blocks). So, each response variables were analyzed using the following model:

y=ijkμ+block+itillage+jblock*tillage+ijsequencek

(1) +tillage*sequence+jkε,ijk

where sequence_*k*_, tillage_*j*_, tillage^∗^sequence_*jk*_ are fixed effects of sequence, tillage treatment and their interaction, respectively. While block_*i*_, block^∗^tillage_*ij*_ and ε_*ijk*_ are random effects of block, main-plot error and sub-plot error (residual). The model was fitted using SAS-software and MIXED-procedure using REML estimation method. Data from the three different depths were analyzed separately.

Assumptions about normal distribution and homogeneity of error variance was checked using box-plots of residuals and scatter plot of residual and fitted values. Some variables of fungistasis activity were normally distributed only after arcsine or square-root transformation. However, all presented estimates were transformed back to the original scale.

Correlation analysis was performed using Pearson’s correlation coefficient if scatter plot of variables showed that the relationship of variables was linear, otherwise Spearman’s rank-order correlation coefficient was used.

Several variables were measured from three different depths in the plot. To test differences in the magnitude of variation in the soil profiles, the coefficient of variation (CV) was calculated for each plot: (standard deviation of observations from three depths)/(mean of observations) × 100%. After that, calculated CV-values were compared statistically using the model applied to other variables.

## Results

### Chemical and Physical Properties of Soil

In the plowed treatment, the values of the chemical and physical variables (Tables 1, 2 and Supplementary Table S1) were relatively even throughout the different top soil layers, whereas both reduced tillage and no-till had steep gradient profiles in most variables having higher values in the surface layers. Deeper in the top soil profile, the differences in mean values were small between treatments.

**TABLE 1:** Test results of the fixed main effects in the generalized linear mixed models for soil chemical and physical properties in soil.

**Management**	**Soil layer**	**Soil analysis**				
	**Depth (cm)**	**pH_H__2__O_**	**EC (10^–4^S cm^–1^)**	**Bulk density (g cm^–3^)**	**N_min_ (mg kg^–1^)**	**P_Acetate_ (mg kg^–1^)**
**Crop sequence**						
Monoculture	0–5	6.49	1.01a	1.14	7.93a	20.20
Crop rotation	0–5	6.49	1.15b	1.11	11.38b	22.41
**Tillage system**						
Plow	0–5	6.45	0.84a	1.11	5.63a	17.01(*a*)
Reduced tillage	0–5	6.56	1.19b	1.13	11.56b	24.73(*b*)
No-till	0–5	6.46	1.21b	1.15	11.78b	22.18(*b*)
**Crop sequence**						
Monoculture	5–10	6.43	0.85	1.28	6.50a	15.85
Crop rotation	5–10	6.37	0.91	1.25	9.36b	17.75
**Tillage system**						
Plow	5–10	6.43a	0.82a	1.22a	6.76a	16.39
Reduced tillage	5–10	6.47a	0.99b	1.29b	9.54b	20.70
No-Till	5–10	6.30b	0.82a	1.30b	7.48a	13.32
**Crop sequence**						
Monoculture	10–20	6.44	0.79	1.35	5.33a	14.67
Crop rotation	10–20	6.39	0.83	1.35	7.56b	15.95
**Tillage system**						
Plow	10–20	6.48	0.83a	1.27a	7.07a	16.65
Reduced tillage	10–20	6.39	0.83a	1.40b	6.57a	16.70
No-till	10–20	6.38	0.76b	1.39b	5.70b	12.57

*The number of observations (*n*) is 24 for each response variable. The different letters refer to statistically significant differences within each comparison; *p* ≤ 0.05, (*p* ≤ 0.10).*

Crop rotation, which had been performed only two growing seasons before the soil sampling, did not markedly affect soil chemical and physical properties, compared to barley monoculture, even though the mean values were consistently higher at crop rotation. The only exception was N_*min*_, where rotation (oats after faba bean) had statistically significantly higher values than monoculture (Table 1), in spite of the reduced mineral fertilizer use for oats.

### Soil Organic Carbon and Microbial Biomass Carbon in the Soil Profile

In no-till and reduced tillage treatments, soil organic carbon (SOC) content decreased by depth and formed a resource gradient for soil micro-organisms (Tables 2, 3). The gradient was especially steep in soil surface (0–5 cm vs. 5–10 cm), and steeper in no-till than in reduced tillage. On average, higher SOC content was detected on no-till (3.36% C) or reduced tillage (3.01% C) surface (0–5 cm) than plowed treatment (2.68% C) (*p* < 0.001). Very similar but even steeper profile was detected in POM-C (Particulate Organic Matter Carbon) values, carbon that is most available for soil microbes (Tables 2, 3). However, the total carbon to nitrogen ratio (C/N) of the soil stayed constant (Tables 2, 3).

**TABLE 2:** Test results of the fixed main effects in the generalized linear mixed models for soil carbon fractions.

**Management**	**Soil layer**	**Soil analysis**			
	**Depth (cm)**	**SOC^a^ (%)**	**C/N**	**POM-C^a^**	**C_mic_^a^ (mg C kg^–1^)**
**Crop sequence**					
Monoculture	0–5	2.99	11.38	69.90	432.4(a)
Crop rotation	0–5	3.05	11.35	72.42	463.2(b)
**Tillage system**					
Plow	0–5	2.68a	11.40	56.76a	347.0a
Reduced tillage	0–5	3.01b	11.46	73.78b	471.4b
No-till	0–5	3.36c	11.23	82.95c	524.9c
**Crop sequence**					
Monoculture	5–10	2.68	11.50	53.85	328.11
Crop rotation	5–10	2.76	11.38	55.48	339.17
**Tillage system**					
Plow	5–10	2.69	11.47	54.99(a)	313.80a
Reduced tillage	5–10	2.75	11.75	56.82(a)	348.25b
No-till	5–10	2.71	11.12	52.18(b)	338.87b
**Crop sequence**					
Monoculture	10–20	2.56	11.28	47.94	301.97
Crop rotation	10–20	2.63	11.38	50.11	307.83
**Tillage system**					
Plow	10–20	2.71a	11.44	54.22a	329.62a
Reduced tillage	10–20	2.48b	11.36	44.47b	281.93b
No-till	10–20	2.59c	11.19	48.37b	303.14c

*^*a*^SOC, soil organic carbon; POM-C, particulate organic matter carbon; C_*mic*_, microbial biomass carbon. The different letters refer to statistically significant differences within each comparison; *p* ≤ 0.05, (*p* ≤ 0.10). The number of observations (*n*) is 24 for each response variable.*

**TABLE 3:** Coefficient of variation (CV) for chemical, physical, and biological variables in the 0–5, 5–10, and 10–20 cm soil layers.

**Variable**	**Plow**	**Reduced tillage**	**No-till**	***p*-value**
pH	0.6a	1.5b	1.5b	<0.01
EC	6.2a	18.1b	25.9c	<0.001
Bulk Density	7.3a	11.0b	9.8(b)	0.03
N_min_	14.0a	27.1b	37.6c	<0.001
P_available_	2.4a	20.4b	34.1c	<0.001
SOC	0.8a	9.6b	14.3c	<0.001
C/N	2.5	3.9	3.1	0.57
POM-C	4.4a	25.3b	31.1c	<0.001
Cmic	6.0a	26.2b	30.7c	<0.001

*The different letters refer to statistically significant differences within each comparison; *p* ≤ 0.05, (*p* ≤ 0.10).*

The microbial biomass carbon (C_*mic*_) content of soil closely followed the vertical distribution of SOC and POM-C (Tables 2, 3). The top 5 cm on reduced and no-till treatments had clearly higher C_*mic*_ values than plowing (*p* < 0.001; Table 3). Crop rotation had the tendency to increase the C_*mic*_ content of soil, as well (*p* < 0.07). The amount of C_*mic*_ separated all tillage treatments from each other both on the top-soil (0-5 cm) and deeper at 10-20 cm (*p* < 0.001). The soil from plowing practice had the most even distribution of C_*mic*_ (CV 6.0%; Table 2), whereas the soil from no-till fields had highest amount of C_*mic*_ on the surface, but the strongest decline deeper in the soil (CV 30.7%). The C_*mic*_ contains a mixture of soil microbial carbon with no separation between e.g., bacterial and fungal origin.

The total amounts of SOC and C_*mic*_ on the topsoil layer were calculated based on both fixed 0–20 cm depth and on the equivalent soil mass method (equivalent mineral soil mass of 200 kg m^−2^, ≈15 cm depth; Wendt and Hauser, 2013; Singh et al., 2015) which takes soil bulk density into account (Table 4). Plowed treatment contained statistically significantly less SOC (6.37 kg C m^−2^) on 20 cm depth compared to the reduced tillage and no-till treatments (6.76 and 7.08 kg C m^−2^; *p* < 0.01, respectively). The difference turned to non-significant with the equivalent soil mass results between plow and reduced tillage (5.24, 5.29, and 5.54 kg Cm^−2^ on plow, reduced tillage and no-till treatments, respectively). Crop rotation did not change SOC in tillage treatments (Table 4). Mean C_*mic*_ of the treatment combinations ranged from 65.3 and 77.0 g C_*mic*_ m^−2^ in the soil layer equivalent to 200 kg m^−2^ (Table 4), which is about 1.1–1.4% of the total soil C stock.

**TABLE 4:** Test results of the fixed main effects in the generalized linear mixed models for soil carbon pools in soil.

Management	Soil carbon pools*			
	
	SOC_20__cm_	SOC_eq_	Cmic_20__cm_	Cmic_eq_
**Crop sequence**				
Monoculture	6.69	5.30	86.4	69.6
Crop rotarion	6.79	5.42	88.6	72.4
**Tillage system**				
Plow	6.37a	5.24a	80.0a	65.9a
Reduced tillage	6.76b	5.29a	88.3b	71.3b
No-till	7.08c	5.54b	94.2c	75.7c

*Soil Organic Carbon (SOC; kg m^−2^) and Microbial Biomass Carbon (Cmic; g m^−2^) in the soil profile either calculated as carbon content on 20 cm top soil or as equivalent soil mass (eq; 200 kg m^−2^; ≈15 cm depth). The number of observations (n) is 24 for each response variable. ^*a*^The different letters refer to statistically significant differences within each comparison; *p*≤0.05.

### Soil Suppressiveness Activity

Fungistasis is an inherent property of natural soil mediated mostly by soil micro-organism and it is interlinked to soil plant pathogen suppressiveness. Soil tillage methods had clear impacts on the soil fungistasis activity in the arable soil. On the top 0-5 cm soil, the plowing treatment had the least suppressive soil against the test fungus *F. culmorum*, whereas reduced and no-till treatments increased the *F. culmorum* fungistasis activity (Table 5). There were no statistically significant differences on fungistasis between the treatments in deeper soil layers below 5 cm (Fung_mm^2^; Supplementary Table S1).

**TABLE 5:** Test results of the fixed main effects in the generalized linear mixed models for fungistasis activity in soil (mm^2^ at the surface bioassay; a smaller area indicates a stronger fungistasis) in the layer of 0-5 cm, and for yield per hectare (kg ha^–1^).

**Management**	**Soil Fungistasis (mm^2^)**	**Yield per Hectare (kg ha^–1^)**
**Crop sequence**		
Monoculture	338.3	4523
Crop rotation	282.6	4968
**Tillage system**		
Plow	363.5a	4684 a
Reduced tillage	284.4b	4440 a
No-till	283.5b	5114 b

*The different letters refer to statistically significant differences within each comparison; *p* ≤ 0.05. The number of observations (*n*) is 24 for each response variable.*

**FIGURE 1:**
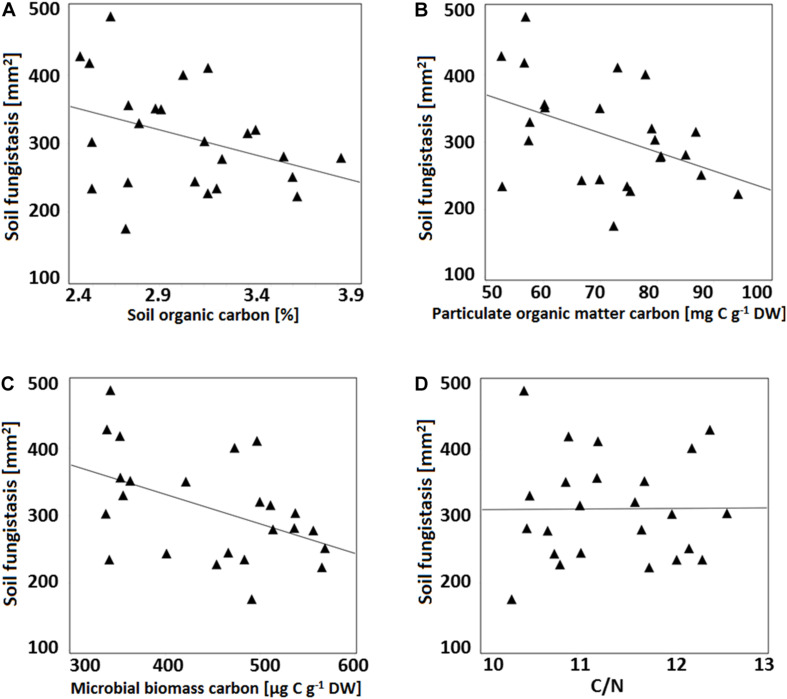
Correlation between fungistasis activity in soil (*y*-axis; mm^2^ at the surface bioassay; the smaller growth area of the test fungus *F. culmorum*, the stronger the soil fungistasis) and different top soil (0–5 cm) organic carbon fractions. **(A)** Soil organic carbon (SOC), %; *r* = –0.34, *p* = 0.10); **(B)** Particulate organic matter carbon (POM-C), mg C g^–1^; *r* = –0.40, *p* = 0.05); **(C)** Microbial biomass carbon (Cmic), μg Cmic g^–1^; *r* = –0.40, *p* = 0.06), and **(D)** C/N (Soil total carbon to nitrogen ratio; *r* = 0.05, *p* = 0.82).

Growth of *F. culmorum* in bioassay correlated negatively with the soil carbon fractions of SOC, POM-C and C_*mic*_ (SOC *r* = −0.34, *p* = 0.10; POM-C: *r* = −0.40, *p* = 0.05; C_*mic*_ *r* = −0.40, *p* = 0.06; Figures 1A–C), that is, the results correlated positively with the fungistasis activity (negative correlation with the growth of test fungus *F. culmorum*). The correlation was strongest with the labile carbon POM-C and weakest with the total SOC. There was no correlation between C/N ratio and the bioassay results (*r* = 0.05, *p* = 0.82; Figure 1D).

### Prevalence of Soil-Borne *Fusarium* spp. in the Field and Crop Yield

**FIGURE 2:**
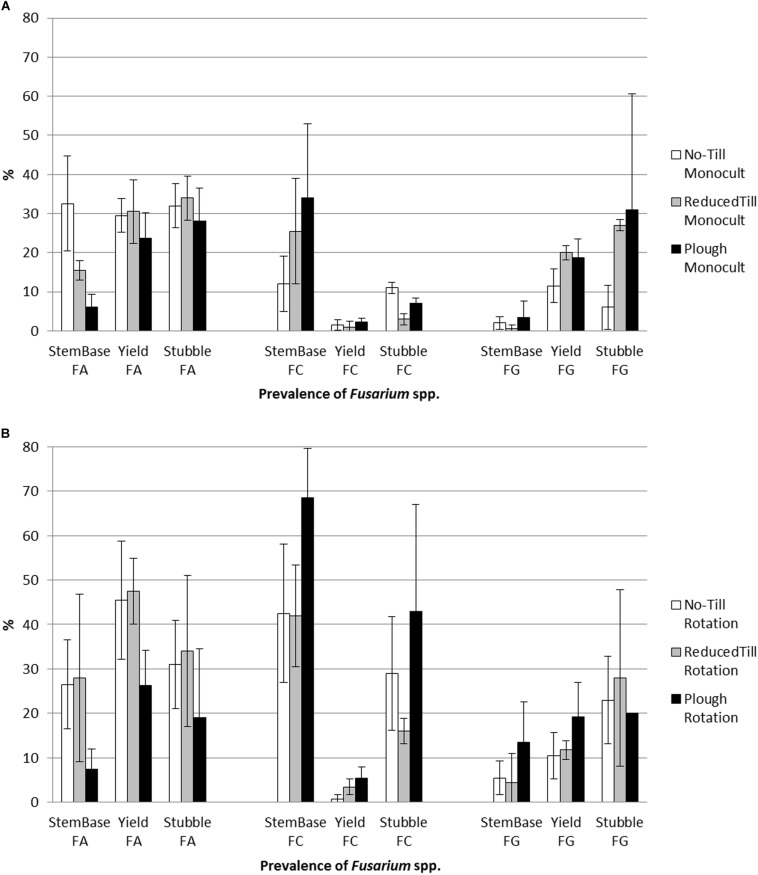
The prevalence of toxins forming pathogens *F. culmorum* (FC) and *F. graminearum* (FG), and predominantly saprophytic species *F. avenaceum* (FA) on tillage treatments in **(A)** barley monoculture and **(B)** oats (crop rotation; oats is known to be more susceptible to *F. culmorum* than barley), on stem base 2 weeks after heading phase, yield during harvest, and at stubble after harvest (% ±SD; mean values, *N* = 4). See Supplementary Table S2 for statistical test results.

During the growing season 2013, repeated samplings were carried out to detect the prevalence of *Fusarium* spp. on cereals growing on the experimental field. The most common species were *F. culmorum, F. graminearum* and *F. avenaceum.* The prevalence of the species in stem base, yield and stubble is shown in Figure 2A (monoculture; barley) and Figure 2B (crop rotation; oats). Generally, oats is seen to be especially susceptive to *Fusarium* spp. pathogen strains in boreal environment (Hofgaard et al., 2016), which was reflected on the results. Crop species at crop sequence (barley monoculture vs. oats at crop rotation) explained the differences in stem base (*p* < 0.01) and stubble (*p* < 0.06).

**FIGURE 3:**
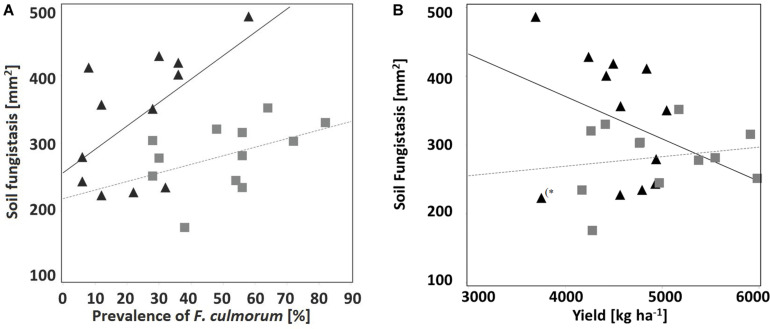
Correlation between fungistasis activity in soil (*y*-axis; mm^2^ at the surface bioassay; the smaller area – the stronger fungistasis) and **(A)** prevalence of test fungus *F. culmorum* (*x*-axis; %) on crop stem base; Triangle = monoculture (*r* = 0.59, *p* = 0.04), Square = rotation (oats), *r* = 0.45, p = 0.15 (all tillage treatments combined), and **(B)** yield (kg ha^–1^); Triangle = monoculture (*r* = –0.29, *p* = 0.35; without an outlier ^(*^r = –0.15, *p* = 0.03), Square = rotation (oats), *r* = 0.18, *p* = 0.58 (all tillage treatments combined).

Non-inversion management decreased the occurrence of the toxins forming pathogens *F. culmorum* and *F. graminearum* whereas the predominantly saprophytic species *F. avenaceum* was detected more often (Figures 2A,B and Supplementary Table S2). On barley monoculture, *F. graminearum* was the main mycotoxin DON producer (Kaukoranta et al., 2019) in grain (harvested and dried) and stubble (collected and analyzed in September) under plowing. The toxins forming pathogen *F. culmorum* was the test fungus for the fungistasis surface assay (see section “Fungistasis Surface Bioassay”). Negative correlation between fungistasis activity in soil and prevalence of test fungus *F. culmorum* on stem base of crop plants was clear under monoculture (*r* = 0.59, *p* = 0.04; Figure 3A).

Crop yield of the experiment in study year 2013 was over the average of the area [Official Statistics of Finland (2020); Supplementary Table S2, and Table 5]. From the tillage treatments, no-till produced the highest yield per hectare (*p* < 0.01), no-till with crop rotation being the best treatment combination. However, there was statistically significant correlation between crop yield and soil fungistasis only in monoculture after removal of an outlier (^∗^*r* = −0.15, *p* = 0.03; Figure 3B).

## Discussion

### General Disease Suppressiveness of Soil

To our knowledge, this was the first study on the impacts of reduced and no tillage combined with crop sequence on the general disease suppressiveness of arable soil in boreal climate. The long-term reduced and no tillage management practices were shown to improve the disease suppressiveness of soil compared to conventional plowing practice. The improved disease suppressiveness was related to the improved labile carbon status, and increased microbial biomass of the soil surface layer. Our findings furthermore demonstrate the correlation of the soil fungistasis bioassay results and the prevalence of the pathogenic test fungus *F. culmorum* on the crop cereals in the field.

Soil-borne plant diseases are among the most important limiting factors for plant productivity in agriculture and difficult to control (Oerke, 2006). Crops lack genetic resistance to most necrotrophic pathogens (Cha et al., 2016). The possibility to improve natural soil disease suppressiveness through agricultural management practices would offer a cost effective and environmental friendly option, and show potential to the sustainable and resilient crop production system (Bailey-Serres et al., 2019). Non-inversion management (reduced and no tillage) with and without crop rotation improved soil fungistasis compared to plowed barley monoculture in boreal climate. Previously, van Agtmaal et al. (2018) suggested that the natural pathogen suppression by volatile compounds produced by soil microbes can be promoted via management. Suppression of the pathogen *Rhizoctonia solani* was most related to the organic matter content of soil, whereas suppression of *Fusarium oxysporum* was driven by field management of reduced tillage. Also, Friberg et al. (2019) found that OTUs (Operational Taxonomic Unit) representing putative plant pathogens *Fusarium culmorum*/*graminearum* were less abundant after non-inversion tillage.

Sharma-Poudyal et al. (2017) concluded that tillage practices have a profound impact on soil fungal communities in agricultural systems. Their results suggest that taxa more common in no-till are more suited to exploit decaying roots as a food source and potentially perform as root endophytes. The fungi would get an advantage in competition for colonizing the dying root. However, another possibility is that tillage is a mechanical disturbance to the fungal populations and hyphal networks, and they are negatively impacted by it. Sipilä et al. (2012) showed that the total microbial biomass was depth dependent in no-till, i.e., no-till accumulated microbial biomass in the surface soil but not in plowed fields and that the strong difference could be seen especially in fungal biomass. Despite that the fungal biomass was not separately measured in the current study, it is very likely that the no-till surface soil, with strongest fungistasis activity, accommodated a microbial consortia with accelerated proportion of saprotrophic fungi (Sipilä et al., 2012; van Agtmaal et al., 2017). Friberg et al. (2019) pointed out that the tillage systems have a significant effect on fungal community already in the first year with non-inversion tillage. The effects on fungal community and crop performance should be considered in relation to the crop sequence used.

Tillage methods have a clear impact on the physical and chemical characteristics of arable soil. If the tillage is reduced, more nutrients and carbon are accumulated on the surface and less is placed to the deeper layers (Muukkonen et al., 2007; Ogle et al., 2019). Our results show that soil conditions for soil microbiome can be improved with reduced mechanical disturbance and increased amount of soil organic carbon, especially labile carbon (POM-C). This leads to improved general soil suppression. However, we did not see strong correlation between SOC content and the soil suppressiveness, which could indicate the importance of SOC quality.

In line with this, Bongiorno et al. (2019b) found that soil suppressiveness was explained by labile carbon and microbial biomass in the soil, but not by the total content of soil organic matter. Labile carbon is important for the maintenance of an abundant and active soil microbiome, essential for the function of suppressive soil. They analyzed several chemical, physical and biological soil quality indicators from the study fields across Europe. Only 25% of the soil suppressiveness could be explained by the soil parameters measured, suggesting that other mechanisms contribute to soil suppressiveness, as well, like the presence and the activity of specific bacterial and fungal taxa with high biocontrol activity.

However, there is accumulating evidence showing the suppressive functions to be of the entire resident soil microbial community, instead of individual, beneficial microbial components (Toyota and Shira, 2018), and that the diversity of microbial taxonomic diversity is not linked to suppressiveness (Bonanomi et al., 2018). In fact, van Agtmaal et al. (2018) showed that only a small portion of natural disease suppression (caused by volatile organic compounds) was explained by microbial community attributes. Soil functionality depends on the community pattern, but not necessary in a direct way (Siegel-Hertz et al., 2018). It is possible that the activity of the microorganisms is directly involved in the targeted function (like disease suppression) only in the presence of certain other community members, without them to be directly involved in the function (Tyc et al., 2014; Williams et al., 2014; Chao et al., 2016). Plants typically lose >21% of all photosynthate through the roots into the soil. In the same time, soil-borne pathogens and pests reduce crop yields by ≥5–60% annually. This is why the plant–microbe interactions in the rhizosphere required for optimal root and soil health is critical to sustainable intensification of agriculture and needs further investigations (Cha et al., 2016).

### Soil Labile and Organic Carbon

The improved disease suppressiveness was related to the improved soil organic carbon status in the top surface soil and depth related soil microbial biomass gradient. Globally, the loss of soil carbon is of major concern and a goal to increase SOC stock at an annual rate of 0.4% per year (or 4 per 1000 initiative) in all land uses has been set (Soussana et al., 2019). In Finland, cultivated mineral soils have lost SOC during the latest decades, relative decrease being 0.4% yr-1. This corresponds to a C stock loss of 220 kg ha-1 yr-1 (equivalent mineral soil mass; Heikkinen et al., 2013). The loss has been strongest in fields of continuous annual crops. Singh et al. (2015) studied the effects of tillage and straw management on soil aggregation and soil carbon sequestration in a 30-year split-plot experiment on clay soil in southern Finland. They concluded that the chances to increase topsoil carbon sequestration by reduced tillage or straw management practices appear limited in cereal monoculture systems of the boreal region.

Our results showed a clear impact of no-tillage and reduced tillage on the organic carbon content and distribution in the arable soil. Even if the total SOC stock did not change, more carbon was concentrated on the surface (0-5 cm) and less carbon was placed to the deeper layers. Similarly, Ogle et al. (2019) concluded based on their extensive literature review that SOC storage can be higher under no-till management in some soil types and climatic conditions, however, uncertainties tend to be large, and no-till may be better viewed as a method for reducing soil erosion and adapting to climate change. It should be noted that SOC improves the water holding capacity of soil, which is an important feature under drought conditions. This could explain that the importance of tillage system on soil suppressiveness may vary depending on the overall soil conditions and activity.

In our study, labile carbon stock, measured both as POM-C and as microbial biomass carbon, was however, higher under no-till. Even if the proportion of microbial biomass carbon is only ca. 1-2% of the total C stock, it is important to notice that the vertical distribution and high concentration on the top surface soil may be enough to cause changes in soil microbial functions.

### Soil Fungistasis and Prevalence of Soil-Borne *Fusarium culmorum* in the Field

Fusarium head blight (FHB) disease, caused by several *Fusarium* species, is a serious threat on cereal yield and grain quality and is expected to benefit from future warmer and more humid climate in boreal area (Parikka et al., 2012; Hofgaard et al., 2016). According to Hofgaard et al. (2016), the increased amount of cereal residues and inoculum potential was thought to cause the increased occurrence of *Fusarium* mycotoxins in Norwegian cereals during the last decades, as a result of increasingly common non-inversion tillage practices. It had been a generally accepted idea that plowing practices were a tool to reduce the potential for *Fusarium* spp. to infect cereals. However, recently, Kaukoranta et al. (2019) concluded from a large survey data of 804 spring-oat fields in Finland that *Fusarium* spp., especially pathogenic *F. culmorum*, tended to be more common under plowing than under non-inversion tillage. In line with this, we found that the predominantly saprophytic *F. avenaceum* was more abundant under non-inversion practice, whereas the opposite was true for the pathogenic ones. Sipilä et al. (2012) linked high fungal biomass with high soil fungistasis activity.

Our field observations confirmed the fungistasis bioassay results: the general plant pathogen suppressiveness of soil could be improved by agricultural management, even if the impact of specific management practice may not be directly and generally linked with the disease suppression activity (Sipilä et al., 2012; Bongiorno et al., 2019b). Our findings furthermore demonstrated the correlation between the laboratory surface assay for fungistasis and the prevalence of the pathogenic test fungus *F. culmorum* on the crop cereals in the field. This makes the laboratory assay a potential tool to estimate the risk of *Fusarium* in cereals. A clear correlation between soil fungistasis and crop yield could not be seen, even though there were indications of correlation especially in monoculture practice. Cereal yields are affected by many different factors and long-term observations would be needed to ensure the connection. However, the no-till management and crop rotation tended to increase the crop yield. The choice of agricultural management practices is proved to be a key for sustainable agricultural production.

## Conclusion

**FIGURE 4:**
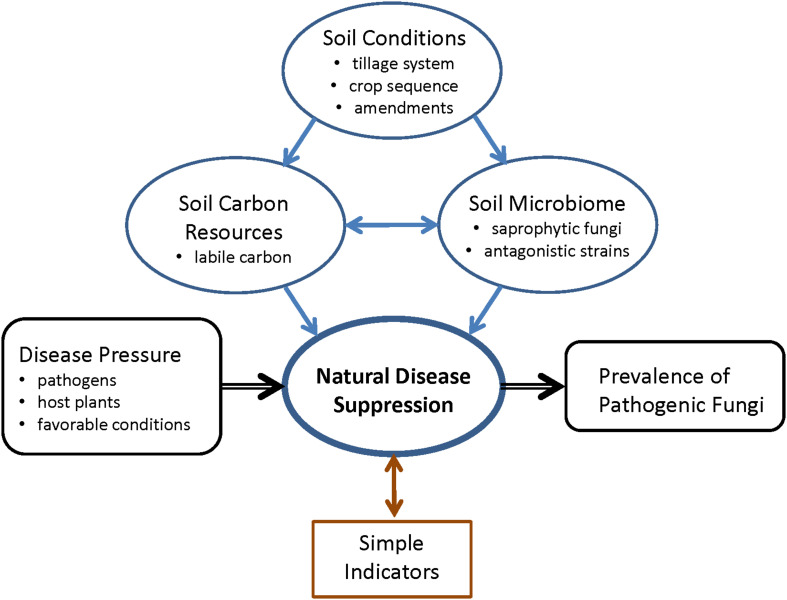
Schematic summary of the study question, and the connections between soil conditions, soil carbon resources, soil microbiome, and natural soil disease suppression.

We demonstrated that agricultural management strategies can be applied to improve the microbial related soil ecosystem functions in the form of natural disease suppressiveness in boreal climate (Figure 4). The conditions for soil microbial communities can be manipulated by the choice of appropriate tillage and crop sequence system. Non-inversion methods, especially the no-tillage management, were shown to change the vertical distribution of carbon fractions and accumulate the SOC, labile carbon and microbial biomass carbon in the soil surface layer. Crop sequence and the choice of tillage method potentially have a combined effect on improved SOC sequestration. General soil disease suppression correlated with labile carbon and microbial biomass carbon, and had a potential impact on crop production, shown as correlation with the prevalence of the test pathogen *F. culmorum* on crops and indications of correlation with yield. The soil surface fungistasis bioassay is potentially a useful tool to monitor general soil suppressiveness. In light of these results, it is crucial to take into consideration the functionality of the whole soil microbiome when planning optimal agricultural management practices.

## Data Availability Statement

All datasets generated for this study are included in the article/Supplementary Material.

## Author Contributions

AP contributed to designing the experiments and writing the manuscript. AP, MK, and PP contributed to collecting the data. AP and LJ contributed to analyzing the data. LA contributed to discussing the design and analyzing the data. All authors reviewed and approved the manuscript before its submission.

## Conflict of Interest

The authors declare that the research was conducted in the absence of any commercial or financial relationships that could be construed as a potential conflict of interest.

## References

[B1] Bailey-Serres J. Parker J. E. Ainsworth E. A. Oldroyd G. E. D. Schroeder J. I. (2019). Genetic strategies for improving crop yields. *Rev. Nat.* 575 109–118. 10.1038/s41586-019-1679-0 31695205 PMC7024682

[B2] Bonanomi G. Cesarano G. Vincenzo A. Di Malo C. De Filippis F. Scala F. (2018). Conventional farming impairs *Rhizoctonia solani* disease suppression by disrupting soil food web. *J. Phytopathol.* 166 663–673. 10.1111/jph.12729

[B3] Bongiorno G. Bünemann E. K. Oguejiofor C. U. Meier J. Gort G. Comans R. (2019a). Sensitivity of labile carbon fractions to tillage and organic matter management and their potential as comprehensive soil quality indicators across pedoclimatic conditions in Europe. *Ecol. Indic.* 99 38–50. 10.1016/j.soilbio.2019.03.012

[B4] Bongiorno G. Postma J. Bünemann E. K. Brussaard L. de Goede R. G. M. Mäder P. (2019b). Soil suppressiveness to *Pythium ultimum* in ten European long-term field experiments and its relation with soil parameters. *Soil Biol. Biochem.* 133 174–187. 10.1016/j.ecolind.2018.12.008

[B5] Box G. E. P. Hunter J. S. Hunter W. G. (2005). *Statistics for Experimenters: Design, Innovation, and Discovery*, 2nd Edn. New York, NY: Wiley, 672.

[B6] Cambardella C. A. Elliott E. T. (1992). Particulate soil organic matter changes across a grassland cultivation sequence. *Soil Sci. Soc. Am. J.* 56 777–783.

[B7] Cha J.-Y. Han S. Hong H.-J. Cho H. Kim D. Kwon Y. (2016). Microbial and biochemical basis of a *Fusarium* wilt-suppressive soil. *ISME J.* 10 119–129. 10.1038/ismej.2015.95 26057845 PMC4681868

[B8] Chao Y. Q. Liu W. S. Chen Y. M. Chen W. H. Zhao L. H. Ding Q. B. (2016). Structure, variation, and co-occurrence of soil microbial communities in abandoned sites of a rare earth elements mine. *Environ. Sci. Technol.* 50 11481–11490. 10.1021/acs.est.6b02284 27670106

[B9] Constanzo A. Barberi P. (2014). Functional agrobiodiversity and agroecosystem services in sustainable wheat production. *A Rev. Agron. Sustain. Dev.* 34 327–348. 10.1007/s13593-013-0178-1

[B10] de Boer W. Gunnewiek P. J. Woldendorp J. W. (1998). Suppression of hyphal growth of soil-borne fungi by dune soils from vigorous and declining stands of *Ammophila arenaria*. *New Phytol.* 138 107–116.

[B11] de Boer W. Li X. Meisner A. Garbeva P. (2019). Pathogen suppression by microbial volatile organic compounds in soils. *FEMS Microbiol. Ecol.* 95:fiz105. 10.1093/femsec/fiz105 31265069

[B12] de Boer W. Wagenaar A. M. Klein Gunnewiek P. J. van Veen J. A. (2007). In vitro suppression of fungi caused by combinations of apparently non-antagonistic soil bacteria. *FEMS Microbiol. Ecol.* 59 177–185. 10.1111/j.1574-6941.2006.00197.x 17233750

[B13] Dignam B. E. A. O’Callaghan M. Condron L. M. Kowalchuk G. A. Van Nostrand J. D. Zhou J. (2018). Effect of land use and soil organic matter quality on the structure and function of microbial communities in pastoral soils: implications for disease suppression. *PLoS One* 13:e0196581. 10.1371/journal.pone.0196581 29734390 PMC5937765

[B14] Ellert B. Bettany J. (1995). Calculation of organic matter and nutrients stored in soils under contrasting management regimes. *Can. J. Soil Sci.* 75 529–538.

[B15] European Commission (2017). *On Member State National Action Plans and on Progress in the Implementation of Directive 2009/128/EC on the Sustainable use of Pesticides.* Report from the Commission to the European Parliament and the Council. COM(2017) 587 final, Brussels: European Commission.

[B16] Friberg H. Persson P. Jensen D. F. Bergkvist G. (2019). Preceding crop and tillage system affect winter survival of wheat and the fungal communities on young wheat roots and in soil. *FEMS Microbiol. Lett.* 366:fnz189. 10.1093/femsle/fnz189 31504475 PMC6759068

[B17] Garbeva P. Hol W. Termorshuizen A. J. Kowalchuk G. A. de Boer W. (2011). Fungistasis and general soil biostasis-A new synthesis. *Soil Biol. Biochem.* 43 469–477. 10.1016/j.soilbio.2010.11.020

[B18] Heikkinen J. Ketoja E. Nuutinen V. Regina K. (2013). Declining trend of carbon in Finnish cropland soils in 1974–2009. *Glob. Chang. Biol.* 19 1456–1469. 10.1111/gcb.12137 23505137

[B19] Hietaniemi V. Rämö S. Yli-Mattila T. Jestoi M. Peltonen S. Kartio M. (2016). Updated survey of *Fusarium* species and toxins in Finnish cereal grains. *Food Addit. Contam. Part A Chem. Anal. Control Expo. Risk Assess.* 33 831–848. 10.1080/19440049.2016.1162112 27002810

[B20] Hofgaard I. S. Seehusen T. Aamor H. U. Riley H. Razzghian J. Le V. H. (2016). Inoculum potential of *Fusarium* spp. relates to tillage and straw management in Norwegian fields of spring oats. *Front. Microbiol.* 7:556. 10.3389/fmicb.2016.00556 27148236 PMC4841101

[B21] IUSS Working Group WRB (2006). *World Reference Base for Soil Resources: A Framework for International Classification, Correlation and Communication.* World Soil Resources Reports 103, Rome: FAO.

[B22] Janvier C. Villeneuve F. Alabouvette C. Edel-Hermann V. Mateille T. Steinberg C. (2007). Soil health through soil disease suppression: which strategy from descriptors to indicators? *Soil Biol. Biochem.* 39 1–23. 10.1016/j.soilbio.2006.07.001

[B23] Kassam A. Friedrich T. Derpsch R. (2019). Global spread of conservation agriculture. *Int. J. Environ. Stud.* 76 29–51. 10.1080/00207233.2018.1494927

[B24] Kaukoranta T. Hietaniemi V. Rämö S. Koivisto T. Parikka P. (2019). Contrasting responses of T-2, HT-2 and DON mycotoxins and *Fusarium* species in oat to climate, weather, tillage and cereal intensity. *Eur. J. Plant Pathol.* 155 93–110. 10.1007/s10658-019-01752-59

[B25] Knudsen I. Debosz K. Hockenhull J. Jensen D. F. Elmholt S. (1999). Suppressiveness of organically and conventionally managed soils towards brown foot rot of barley. *Appl. Soil Ecol.* 12 61–72.

[B26] Laine M. Rütting T. Alakukku L. Palojärvi A. Strömmer R. (2018). Process rates of nitrogen cycle in uppermost topsoil after harvesting in no-tilled and ploughed agricultural clay soil. *Nutr. Cycl. Agroecosyst.* 110 39–49. 10.1007/s10705-017-9825-22

[B27] Legrand F. Chen W. Cobo-Diaz J. F. Picot A. Le Floch G. (2019). Co-occurrence analysis reveal that biotic and abiotic factors influence soil fungistasis against *Fusarium graminearum*. *FEMS Microbiol. Ecol.* 95 1–13. 10.1093/femsec/fiz056 30998232

[B28] Lockwood J. L. (1977). Fungistasis in soils. *Biol. Rev.* 52, 1–43. 10.1111/j.1469-185X.1977.tb01344.x

[B29] Mendes R. Kruijt M. de Bruijn I. Dekkers E. van der Voort M. Schneider J. H. M. (2011). Deciphering the rhizosphere microbiome for disease-suppressive bacteria. *Science* 332 1097–1100. 10.1126/science.1203980 21551032

[B30] Muukkonen P. Hartikainen H. Lahti K. Särkelä A. Puustinen M. Alakukku L. (2007). Influence of no-tillage on the distribution and lability of phosphorus in clay soils. *Agric. Ecol. Environ.* 120 299–306. 10.1016/j.agee.2006.09.012

[B31] Nelson P. E. Toussoun T. A. Marasas W. F. O. (1983). *Fusarium Species: An Illustrated Manual for Identification.* Pennsylvania, PA: Pennsylvania State University Press.

[B32] Oerke E.-C. (2006). Crop losses to pests. *J. Agric. Sci.* 144 31–43. 10.1017/S0021859605005708

[B33] Official Statistics of Finland (2020). *Yield of the Main Crops.* Available online at: http://stat.luke.fi/en/crop-production-statistics (accessed September 27, 2020).

[B34] Ogle S. M. Alsaker C. Baldock J. Bernoux M. Breidt F. J. McConkey B. (2019). Climate and soil characteristics determine where no-till management can store carbon in soils and mitigate greenhouse gas emissions. *Sci. Rep.* 9:11665. 10.1038/s41598-019-47861-7 31406257 PMC6691111

[B35] Parikka P. Hakala K. Tiilikkala K. (2012). Expected shifts in *Fusarium* species’ composition on cereal grain in Northern Europe due to climatic change. *Food Addit. Contam. Part A Chem. Anal. Control Expo. Risk Assess.* 29 1543–1555. 10.1080/19440049.2012.680613 22554046

[B36] Penton C. R. Gupta V. V. S. R. Tiedje J. M. Neate S. M. Ophel-Keller K. Gillings M. (2014). Fungal community structure in disease suppressive soils assessed by 28S LSU gene sequencing. *PLoS One* 9:e93893. 10.1371/journal.pone.0093893 24699870 PMC3974846

[B37] Postma J. Schilder M. T. Bloem J. van Leeuwen-Haagsma W. K. (2008). Soil suppressiveness and functional diversity of the soil microflora in organic farming systems. *Soil Biol. Biochem.* 40 2394–2406. 10.1016/j.soilbio.2008.05.023

[B38] Prestele R. Hirsch A. L. Davin E. L. Seneviratne S. I. Verburg P. H. (2018). A spatially explicit representation of conservation agriculturefor application in global change studies. *Glob. Chang. Biol.* 24 4038–4053. 10.1111/gcb.14307 29749125 PMC6120452

[B39] Regina K. Alakukku L. (2010). Greenhouse gas fluxes in varying soils types under conventional and no-tillage practices. *Soil Tillage Res.* 109 144–152. 10.1016/j.still.2010.05.009

[B40] Sharma-Poudyal D. Schlatter D. Yin C. Hulbert S. Paulitz T. (2017). Long-term no-till: a major driver of fungal communities in dryland wheat cropping systems. *PLoS One* 12:e0184611. 10.1371/journal.pone.0184611 28898288 PMC5595340

[B41] Siegel-Hertz K. Edel-Hermann V. Chapelle E. Terrat S. Raaijmakers J. M. Steinberg C. (2018). Comparative microbiome analysis of a *Fusarium* wilt suppressive soil and a *Fusarium* wilt conducive soil from the Châteaurenard Region. *Front. Microbiol.* 9:568. 10.3389/fmicb.2018.00568 29670584 PMC5893819

[B42] Singh P. Heikkinen J. Ketoja E. Nuutinen V. Palojärvi A. Sheehy J. (2015). Tillage and crop residue management methods had minor effects on the stock and stabilization of topsoil carbon in a 30-year field experiment. *Sci. Total Environ.* 518-519 337–344. 10.1016/j.scitotenv.2015.03.027 25770946

[B43] Sipilä T. P. Yrjälä K. Alakukku L. Palojärvi A. (2012). Cross-site soil microbial communities under tillage regimes: fungistasis and microbial biomarkers. *Appl. Environ. Microbiol.* 78 8191–8201. 10.1128/AEM.02005-12 22983972 PMC3497356

[B44] Soussana J.-F. Lutfalla S. Ehrhardt F. Rosenstock T. Lamanna C. Havlík P. (2019). Matching policy and science: rationale for the ‘4 per 1000 - soils for food security and climate’ initiative. *Soil Tillage Res.* 188 3–15. 10.1016/j.still.2017.12.002

[B45] Toyota K. Shira S. (2018). Growing interest in microbiome research unraveling disease suppressive soils against plant pathogens. *Microbes Environ.* 33 345–347. 10.1264/jsme2.ME3304rh 30606975 PMC6307993

[B46] Tyc O. van den Berg M. Gerards S. van Veen J. A. Raaijmakers J. M. de Boer W. (2014). Impact of interspecific interactions on antimicrobial activity among soil bacteria. *Front. Microbiol.* 5:567. 10.3389/fmicb.2014.00567 25389421 PMC4211544

[B47] van Agtmaal M. Straathof A. Termorshuizen A. Teurlincx S. Hundscheid M. Ruyters S. (2017). Exploring the reservoir of potential fungal plant pathogens in agricultural soil. *Appl. Soil Ecol.* 121 152–160. 10.1016/j.apsoil.2017.09.032

[B48] van Agtmaal M. Straathof A. L. Termorshuizen A. Lievens B. Hoffland E. de Boer W. (2018). Volatile-mediated suppression of plant pathogens is related to soil properties and microbial community composition. *Soil Biol. Biochem.* 117 164–174. 10.1016/j.soilbio.2017.11.015

[B49] Vuorinen J. Mäkitie O. (1955). *The Method of Soil Testing in use in Finland*, Vol. 63. Helsinki: Valtioneuvoston kirjap, 1–44.

[B50] Wang L. Li X. (2019). Steering soil microbiome to enhance soil system resilience. *Crit. Rev. Microbiol.* 45 5–6. 10.1080/1040841X.2019.1700906 31833440

[B51] Weller D. M. Raaijmakers J. M. Gardener B. B. M. S. Thomashow L. S. (2002). Microbial populations responsible for specific soil suppressiveness to plant pathogens 1. *Annu. Rev. Phytopathol.* 40 309–348. 12147763 10.1146/annurev.phyto.40.030402.110010

[B52] Wendt J. Hauser S. (2013). An equivalent soil mass procedure for monitoring soil organic carbon in multiple soil layers. *Eur. J. Soil Sci.* 64 58–65. 10.1111/ejss.12002

[B53] Williams R. J. Howe A. Hofmockel K. S. (2014). Demonstrating microbial co-occurrence pattern analyses within and between ecosystems. *Front. Microbiol.* 5:358. 10.3389/fmicb.2014.00358 25101065 PMC4102878

