# Corrigendum: Tillage System and Crop Sequence Affect Soil Disease Suppressiveness and Carbon Status in Boreal Climate

**DOI:** 10.3389/fmicb.2021.693341

**Published:** 2021-05-20

**Authors:** Ansa Palojärvi, Miriam Kellock, Päivi Parikka, Lauri Jauhiainen, Laura Alakukku

**Affiliations:** ^1^Natural Resources Institute Finland (Luke), Turku, Finland; ^2^Natural Resources Institute Finland (Luke), Jokioinen, Finland; ^3^Department of Agricultural Sciences, University of Helsinki, Helsinki, Finland

**Keywords:** fungistasis, no-till, non-inversion, *Fusarium* spp., microbial biomass, general disease suppression, crop rotation, labile carbon

In the original article, there was a mistake in Table 4 as published. Inadvertently, a misordered data table was used for calculating SOC (Soil Organic Carbon) pool results for Table 4. The numerical values were slightly erroneous and some of the letters referring to statistically significant differences in each comparison of SOC pools were incorrect. The corrected Table 4 is shown below.

**TABLE 4:** Test results of the fixed main effects in the generalized linear mixed models for soil carbon pools in soil.

**Management**	Soil carbon pools^*^			
	**SOC_**20 cm**_**	**SOC_**eq**_**	**Cmic_**20 cm**_**	**Cmic_**eq**_**
**Crop sequence**				
Monoculture	6.69	5.30	86.4	69.6
Crop rotarion	6.79	5.42	88.6	72.4
**Tillage system**				
Plow	6.37 a	5.24 a	80.0 a	65.9 a
Reduced tillage	6.76 b	5.29 a	88.3 b	71.3 b
No-till	7.08 c	5.54 b	94.2 c	75.7 c

* *Soil Organic Carbon (SOC; kg m^−2^) and Microbial Biomass Carbon (Cmic; g m^−2^) in the soil profile either calculated as carbon content on 20 cm top soil or as equivalent soil mass (eq; 200 kg m^−2^; ≈15 cm depth). The number of observations (n) is 24 for each response variable*.

*^a^The different letters refer to statistically significant differences within each comparison; p ≤ 0.05*.

Consequently, a correction has been made to Results, sub-section ‘Soil Organic Carbon and Microbial Biomass Carbon in the Soil Profile.' The corrected third paragraph is shown below.

The total amounts of SOC and C_*mic*_ on the topsoil layer were calculated based on both fixed 0–20 cm depth and on the equivalent soil mass method (equivalent mineral soil mass of 200 kg m^−2^, ≈15 cm depth; Wendt and Hauser, 2013; Singh et al., 2015) which takes soil bulk density into account (Table 4). Plowed treatment contained statistically significantly less SOC (6.37 kg C m^−2^) on 20 cm depth compared to the reduced tillage and no-till treatments (6.76 and 7.08 kg C m^−2^; *p* < 0.01, respectively). The difference turned to non-significant with the equivalent soil mass results between plow and reduced tillage (5.24, 5.29, and 5.54 kg C m^−2^ on plow, reduced tillage and no-till treatments, respectively). Crop rotation did not change SOC in tillage treatments (Table 4). Mean C_*mic*_ of the treatment combinations ranged from 65.3 and 77.0 g C_*mic*_ m^−2^ in the soil layer equivalent to 200 kg m^−2^ (Table 4), which is about 1.1–1.4% of the total soil C stock.

The authors apologize for this error and state that this does not change the scientific conclusions of the article in any way. The original article has been updated.
